# Medicinal herbs and multiple sclerosis: Overview on the hard balance between new therapeutic strategy and occupational health risk

**DOI:** 10.3389/fncel.2022.985943

**Published:** 2022-11-10

**Authors:** Erica Costantini, Eva Masciarelli, Laura Casorri, Marco Di Luigi, Marcella Reale

**Affiliations:** ^1^Department of Medicine and Science of Aging, G. d’Annunzio University of Chieti–Pescara, Chieti, Italy; ^2^Department of Technological Innovations and Safety of Plants, Products and Anthropic Settlements, National Institute for Insurance Against Accidents at Work, Rome, Italy; ^3^Department of Occupational and Environmental Medicine, Epidemiology and Hygiene, INAIL Research Center, National Institute for Insurance Against Accidents at Work, Rome, Italy; ^4^Department of Innovative Technologies in Medicine and Dentistry, G. d’Annunzio University of Chieti–Pescara, Chieti, Italy

**Keywords:** multiple sclerosis, herbal compounds, medicinal plant, inflammation, pesticides

## Abstract

Multiple sclerosis (MS) is an autoimmune disease characterized by demyelination and axonal loss of the central nervous system (CNS). Despite its spread throughout the world, the mechanisms that determine its onset are still to be defined. Immunological, genetic, viral, and environmental factors and exposure to chemicals may trigger MS. Many studies have highlighted the anti-inflammatory and anti-oxidant effects of medicinal herbs, which make them a natural and complementary treatment for neurodegenerative diseases. A severe reduction of several MS symptoms occurs with herbal therapy. Thus, the request for medicinal plants with potential beneficial effects, for MS patients, is constantly increasing. Consequently, a production increase needs. Unfortunately, many medicinal herbs were untested and their action mechanism, possible adverse effects, contraindications, or interactions with other drugs, are poorly or not investigated. Keeping in mind the pathological mechanisms of MS and the oxidative damages and mitochondrial dysfunctions induced by pesticides, it is important to understand if pesticides used to increase agricultural productivity and their residues in medicinal plants, may increase the risk of developing MS in both workers and consumers. Studies providing some indication about the relationship between environmental exposure to pesticides and MS disease incidence are few, fragmentary, and discordant. The aim of this article is to provide a glance at the therapeutic potential of medicinal plants and at the risk for MS onset of pesticides used by medicinal plant growers and present in medicinal herbs.

## Introduction

Multiple sclerosis (MS) occurs when, in the brain and spinal cord, the immune system attack nerve fibers and myelin sheathing, causing communication difficulties between the brain and the other body areas (Filippi et al., 2018). Researchers reported that a combination of immunological, genetic, viral, and environmental factors and exposure to chemicals may trigger MS (Olsson et al., 2017; Dobson and Giovannoni, 2019). To date, there are no treatments to cure MS, but disease progression control and symptom ease can be obtained with several therapeutic strategies (Ben-Zacharia, 2011; Hauser and Cree, 2020). Thus, among people with MS herbal drugs and dietary supplements use is increasing.

Many studies reported the therapeutic effects of medicinal plants, due to their anti-inflammatory, antioxidant, and myelin-repairing properties, in different disorders such as cancers, diabetes, and neurodegenerative diseases (Piao and Liang, 2012; Mohajeri et al., 2015; Costantini et al., 2022). The efficacy of plants and their active components was then described in patients with MS and in experimental models of mice with autoimmune encephalomyelitis (EAE), through the regulation of immune cells, immune factors, and oxidative stress.

A lot of patients from countries that have a specific cultural bent consider herbal drugs better than conventional drugs because of their natural origin. Recently, even in industrialized countries, herbal drug use has increased because they are considered safer than allopathic drugs. Currently, there are a large number of traditional medicines (TM) and other herbal complementary and alternative (CAM) products available in the whole world as therapeutic agents.

The medicinal plants demand with potential beneficial effects for MS patients, steadily increases requiring their greater production and, consequently, pesticides use increases. Their application during the production or storage of agricultural products leads to pesticide residues in food. This non-occupational exposure to pesticide residues, and their conversion products, metabolites, reaction products, and impurities raise a health risk. Therefore, it becomes more and more important to understand whether pesticides may increase the developing MS risk in farmers.

Rat and mouse animal models, developed to study the pesticide exposure effects, especially the neurotoxic effects, have supported the epidemiological link between Parkinson’s disease (PD) and agricultural pesticide use. Human studies report that exposure to relatively low doses of pesticides used in agriculture may hit brain cells and cause neuron loss in particular regions of the brain, by free radical production induction or lipidic peroxidation, with subsequent cognitive decline, memory, and attention impairment, and motion function deterioration (Sánchez-Santed et al., 2016; Yan et al., 2016; Kori et al., 2018; Naughton and Terry, 2018).

Very few studies have investigated the effects of exposure to pesticides on MS, and the results are fragmentary and discordant. However, a high incidence of MS has been found in the major medical herb-producing countries, and it is increasing, with many cases in adolescence (Hashem et al., 2010; Vasconcelos et al., 2016; Azami et al., 2019). These countries make high use of pesticides whose residues and metabolites are found in food and supplements (Rodrigues et al., 2007; Farag et al., 2011; Sarkhail et al., 2012).

**FIGURE 1:**
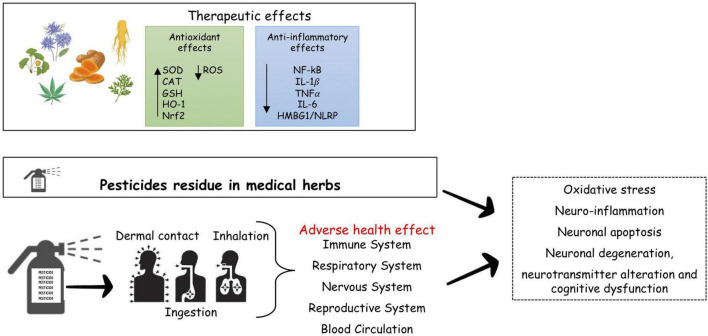
Pesticides and risk of neurodegenerative diseases. Many medical herbal have therapeutic effects in MS, reducing inflammation and oxidative stress, the major driver of MS. Pesticides both occupational pesticides exposure through agricultural activities, and consumers exposure to residual pesticide present in food and herbal medicines, may represent a risk for brain damage, cognitive deficits, behavioral and neurodegeneraive disorders as results of dysregulation of molecular target. SOD, superoxide dismutase; CAT, catalase; GSH, glutathione; HO-1, heme oxygenate-1; Nrf2, nuclear factor E2-related factor-2; NF-kB, nuclear factor k-light-chain-enhancer of activated B cells; IL, interleukin; TNF, tumor necrosis factor; HMBG1/NLRP, high mobility group box1/nucleotide-binding oligomerization domain, leucine rich repeat and pyrin domain containing.

The focus of this manuscript is to provide an overview of the potential efficacy of medicinal herbs in MS and of the risk of developing MS for the customary user, due to the presence of pesticide residues in herbs, or for the farmer due to the use of pesticides in cultivation (Figure 1).

### Step by step

In undertaking this task, preliminary we described the pathophysiology of MS, next we describe the therapeutic efficacy of the main medicinal herbal in MS. Then, we analyzed the safety assessment and international regulation of quality and transparency along the supply chain of medicinal herbs as well as pathogenic mechanisms of pesticides in neurodegeneration and MS.

Finally, we provide a synopsis of where the field is heading, the necessary legislation and progress of appropriate and standardized analytical methods for the detection and identification of pesticides in medicinal herbs to provide novel therapeutics strategies based on medicinal herbs.

### Search strategy

We searched PubMed Medline, Scopus and Research Gate for all studies published between January 1992 to now, using multiple combinations of keywords: pesticides, MS and neurodegeneration. Examining the references of important studies, we also identified potentially relevant citations not recovered by the initial literature searches.

### Study selection

After removing duplicate records, titles and abstracts of literature search results were scanned for eligibility by two reviewers, identified carefully through manual review and discrepancies resolved by a third reviewer. Relevant *in vivo*, *ex vivo*, and *in vitro* studies were selected if presented data from an analytical observational epidemiological study (i.e., cohort, cross-sectional, or case–control study) or data on environmental or occupational exposure to pesticides. The selected studies are heterogeneous in study design and methods, therefore the results cannot be combined through meta-analysis. Therefore, we conducted a narrative synthesis to highlight the state of knowledge on the effects of pesticides on the risk of MS onset.

## Pathophysiology of multiple sclerosis

Multiple sclerosis is a complex pathology with continuous inflammatory activity within the CNS (Central Nervous System), giving rise to tissue damage that results in brain and spinal cord atrophy and irreversible neurological disability. MS affects over 2.5 million people worldwide, especially women and young, usually with an age of onset between 18 and 40 years, seriously reducing the life quality and causing a significant financial burden (Walton et al., 2020).

Despite the longtime of MS investigations, the etiology is still very far from complete understanding, although genetic, epigenetic, and environmental factors seem to be correlated to MS pathogenesis (Oddone and Imbriani, 2015). Numerous studies have identified more than 200 MS genetic risk variants, many of which encode molecules involved in immune system responses, confirming that MS is an immune-mediated disease (Wingerchuk et al., 2001; International Multiple Sclerosis Genetics Consortium [IMSGC], 2013; Baranzini and Oksenberg, 2017; Parnell and Booth, 2017; Dashti et al., 2020). Cholinergic system dysfunction driving an impaired control of inflammation has been associated with BChE and AChE genetic variations in MS patients (Di Bari et al., 2016; Reale et al., 2018).

Low vitamin D levels, smoking, obesity, stress, gut microbiota, infections, immunization, heavy metals, and pesticide exposure have been considered risk factors for MS development (Cannon and Greenamyre, 2011; Chen et al., 2016).

Multiple sclerosis is characterized by a diversified clinical course, generally starting with focal inflammatory events, followed by remissions and relapses. Patients with MS showed heterogeneous symptoms including changes in vision, sensory loss, weakness, cognitive change, and mood disturbance (Lassmann, 2019).

Most MS patients, over time and aging, switch to a progressive phase characterized by a gradual clinical decline with progressive deterioration of neurological functions, due to continuous axonal degeneration (Filippi et al., 2018). The disease progression may lead to severe disability representing a significant limitation of employment and productivity.

The mechanism of MS pathology involves complex interactions between systems and cell types, such as neurons, glia, and immune cells, which lead to focal lesions of inflammatory demyelination and widespread neurodegeneration in the brain and spinal cord. Post-mortem examinations of MS patient’s brains showed typical inflammatory T cells, and macrophages perivascular infiltrates, along with myelin degradation and axons degeneration, but demyelinated lesions can occur anywhere within the brain and spinal cord, giving to the disease complexity, and heterogeneity of clinical signs, and symptoms (Kuhlmann et al., 2008). Autoreactive T helper (Th) cells are considered responsible for the initiation and maintenance of autoreactivity to CNS myelin and are supported by macrophages, B-cells, Natural Killer (NK) cells, cytotoxic T-cells, and microglial cells are key players in MS disease progression (Kaskow and Baecher-Allan, 2018; Guerrero and Sicotte, 2020; Mimpen et al., 2020). Like Th1 cells, also Th17 cells promote inflammation in MS, transmigrating across Blood-Brain Barrier (BBB) and may induce demyelination and axonal loss (Moser et al., 2020). These cells release IFNγ, TNF-α, IL-17, IL-21, IL-22, and IL-26 cytokines, which mediate inflammatory responses (Guerrero and Sicotte, 2020). Especially, the release of IL-17, by Th17, triggers the expression of inducible nitric oxide synthase (iNOS) and concomitant increase of nitric oxide (NO) in various cell types and activates many other inflammatory mediators (Balasa et al., 2020). Oxidative activation in microglia is associated with massive mitochondrial injury and gene deletion in MS lesions with active demyelination or neurodegeneration, in a self-amplifying vicious cycle of microglial activation–induced oxidative stress and mitochondrial injury (Mahad et al., 2008).

Moreover, the function of T regulatory (Treg) cells in MS patients is reduced, and the CD8 + T cells presence can promote vascular permeability and activate oligodendrocyte death (Calahorra et al., 2022). B-cells also play a role in MS, probably by producing soluble neurotoxic factors leading to demyelination and neurodegeneration (Lassmann, 2019).

**FIGURE 2:**
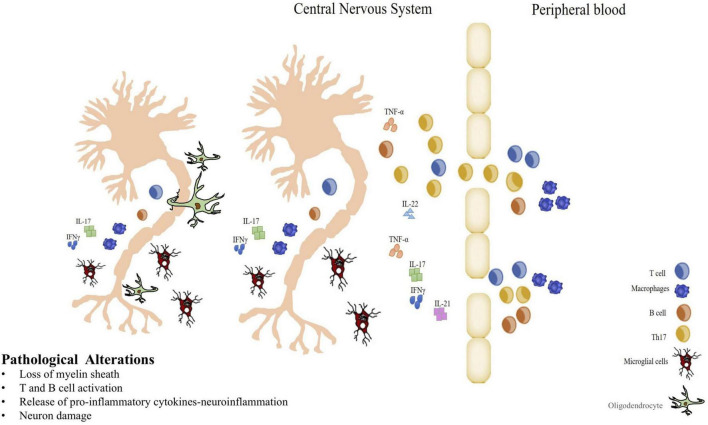
Immunopathogenesis of MS. Blood Brain Barrier breakdown, autoreactive cell infiltration and inflammatory cytokines production in CNS. Myelin-specific T cells entering in the central nervous system (CNS) initiate an inflammatory response, and B cells participate in myelin breakdown secreting antibodies. Resident CNS microglial cells have the capacity to secrete proinflammatory molecules that can damage the myelin sheath and/or oligodendrocytes. When oligodendrocytes are damaged and myelin that typically isolates the axons of nerve cells is lost, demyelination process is in progress playing a key role in MS. Thus, strategies that reduce inflammation may be very influential in altering the pathogenic process in MS.

Many studies’ results point to critical roles for cytokines that drive the inflammation in the brain, attracting more immune cells, causing tissue damage, and the loss of neurological function (Reale et al., 2012; Kallaur et al., 2013; D’Angelo et al., 2018; Bai et al., 2019). The role of inflammatory events mediated by autoreactive T cells in the CNS is broadly shared, although there isn’t a unanimously accepted model for MS (Stys and Tsutsui, 2019). Also, microglia, involved in cross-talk with other immune cells, secrete proinflammatory molecules and may take part in the damage of the myelin sheath and/or oligodendrocytes. Therefore, the damage of the oligodendrocytes and demyelinization was considered the consequence of the infiltration of inflammatory cells and activation of the inflammatory process (Figure 2).

Unfortunately, to date there’s no cure for MS. However, MS therapy has been enriched with a variety of therapeutic approaches to modify the disease course, progression, and control of symptoms (Polman et al., 2006; Gasperini et al., 2013; Yadav et al., 2014; Bergamaschi et al., 2016; Ancau et al., 2019). Alongside the symptomatic therapies to limit discomfort, there are rehabilitation and occupational therapies, equally essential for quality of life (QoL) recovery. Over the last decades, the use of complementary and alternatively herbal remedies, due to their anti-inflammatory, immunomodulatory, and antioxidative characteristics, has noticeably risen in MS patients (Yadav et al., 2010; Dayapoglu and Tan, 2016; Kim et al., 2018).

## Herbal products with therapeutic efficacy in multiple sclerosis

Herbal products are natural formulations, made from whole medicinal plants, or from their parts and their derivatives, not containing synthetic or semi-synthetic products. The definition “*piante officinali*” is exclusively an Italian definition, originating from the term “*opificina*,” which identified the place where the plants were used to produce preparations for therapeutic use, food, liquor, food supplements, cosmetics, medical devices, drugs, etc. The herbal products use continues to expand rapidly around the world. According to the WHO, between 35,000 and 70,000 plant species are worldwide used for various functional purposes but studies have been carried out on only 5,000 of them.

To date, herbal drug regulation, differ across countries (Bhat et al., 2019), and regulatory authorities and the scientific community are working to evolve regulations of herbal medicines more effectively and convergence of different environments. In Europe, these products are instead called using the terms botanical or herbal. The European legislation is very articulate and includes Directives and Regulations concerning both food and medicinal products for human use.

Several studies, conducted mainly in East Asia, have shown that several plants and their compounds have positive effects on MS (Yadav et al., 2010; Dayapoglu and Tan, 2016; Kim et al., 2018). As reported by Peng et al. (2021) there are many prescriptions for Chinese herbal medicine (CHM) to treat MS/experimental autoimmune encephalomyelitis (EAE), while there isn’t enough information about the efficacy of CHM for MS patients.

This review presents a brief summary of the most commonly used herbs and the mechanisms that make them effective in treating MS patients (Table 1).

**TABLE 1:** Summary of herbal medicines with therapeutic effects on MS patients and EAE models.

Medicinal plant	Effects on MS patients and EAE models
*Artemisia dracunculus*	**Reduce** clinical disease score, **lower** cell infiltration, **recover** brain demyelination, **reduce** IL-23 and IL-17A production and **increase** antioxidant efficacy in EAE model (Safari et al., 2021)
*Curcuma longa*	**Reduce** MS clinical severity, **reduce** IL-17, TGF-β, IFN-γ, IL-6, IL-21, IL-23, STAT3, RORγt, **decrease** Th17 infiltration and differentiation in EAE model (Xie et al., 2009) **Modulate** CD4 + T cells, TLRs in EAE model (Chearwae and Bright, 2008) **Decrease** astrocytes proliferation in EAE model (Xie et al., 2011) **Improve** myelinogenesis in EAE model (Mohammadi et al., 2021) **Increase** the oligodendrocytes activity and differentiation in EAE model (Eghbaliferiz et al., 2020) **Reduce** number of TUNEL(+) cells, **reduce** CASP3, Asp353, Cyt-c expression, **increase** MBP expression in the EAE model (Feng et al., 2014) **Inhibit** IL-12 signaling through the JNK-STAT pathway, **reduce** IL-12 production, **decrease** differentiation of neural Ag-specific Th1 cells in EAE model (Natarajan and Bright, 2002) **Reduce** IL-12 production and **increase** IL-10 in PBMC from MS patients (Fahey et al., 2007)
*Ginkgo biloba*	**Improve** memory and QoL in MS patients (Noroozian et al., 2011) **Reverse** cognitive impairment with a moderate beneficial effect in MS patients (Johnson et al., 2006) **Improve** memory and QoL, **reduce** inflammation and fatigue, **reverse** cognitive impairment in MS patients (Lovera et al., 2007) **Ineffective** on cognitive performance in MS patients (Lovera et al., 2012) **Inhibit** TLR4, NF-κB, iNOS, IL-1β, TNF-α expression, **up-regulate** Arg-1, NTF, **regulate** microglia and astrocytes balance, **induce** OPCs generation, myelin production in CPZ-induced demyelinating model (Yin et al., 2020)
*Cannabis sativa*	**Improve** spasticity, neuropathic pain, urinary complications, sleep disorders in MS patients (Greenberg et al., 1994; Pertwee, 2002; Baker and Pryce, 2008) **Suppress** IFN-γ, IL-17, IL-6, IL-1β and TNF-α production in EAE model (Al-Ghezi et al., 2019) **Reduce** TNF-α, **enhance** BDNF production, **improve** neurological disability score in EAE model (Zhou et al., 2019) **Reduce** disease severity, **suppress** IFN-γ producing CD8 + T cells in EAE model (Nichols et al., 2021) **Increase** MDSCs, **reduce** CXCL9, CXCL10, IL-1β, IL-17, IFN-γ expression, **diminish** T cell, macrophages infiltration in EAE model (Elliott et al., 2018; Dopkins et al., 2021) **Modulate** expression of the inflammatory genes, **reduce** disease symptoms in EAE model (Yang et al., 2019)
*Nigella sativa*	**Improve** clinical manifestations, **decrease** reactive astrocytes number, **favor** complete remyelination in EAE model (Fahmy et al., 2014) **Ameliorate** chronic relapsing, **counteract** mononuclear cells infiltration in the brain and spinal cord, **increase** GSH, **inhibit** NF-kB activation in EAE model (Mohamed et al., 2005) **Reduce** TGF-β1 expression, **promote** remyelination, **increase** GSH levels modulating the oxidative balance (Noor et al., 2015)
*Panax ginseng*	**Improve** QoL, **reduce** level of fatigue in MS patients (Etemadifar et al., 2013) **Safe** but **ineffective** for treating fatigue in MS patients (Kim et al., 2011) **Induce** Treg generation, Th1 and Th17 cells suppression, **decrease** infiltration of inflammatory cells, **modulate** CD4 + T cells and CD11b + macrophages infiltration, **increase** Tregs and Foxp3 expression in EAE model (Hwang et al., 2011) **Downregulate** p38 MAPK/NF-κB signaling pathway in EAE model (Lee et al., 2016) **Reduce** demyelination and axonal damages, **reduce** the pro-inflammatory factors production (IFN-γ, IL-17, TNF-α) in EAE model (Bing et al., 2016) **Protect** spinal demyelination, **downregulate** p38 MAPK/NF-κB signaling pathway in EAE model (Lee et al., 2018) **Reduce** disease severity, **decrease** BBB permeability, **endorse** Th2 shift in EAE model (Zhu et al., 2014) **Inhibit** TNF-α circulating levels, iNOS in EAE model (Bowie et al., 2012)
*Zingiber officinale*	**Inhibit** iNOS and COX-2 expression in macrophages, **reduce** apoptosis rate in C57BL/6 murine model (Ali et al., 2008) **Reduce** clinical disease score, **delay** symptoms onset, **reduce** IL-17, IL-27, IL-33 expression, **reduce** infiltration of the inflammatory cells in CNS in EAE model (Jafarzadeh et al., 2014, 2015) **Reduce** expression of IL-33, IL-27, IL-12, IL-27, IL-17 in CNS, **downregulate** CCL20, CCL22, CCR6, CCR4, **increase** TGF-β expression, **inhibit** leukocytes infiltration in EAE model (Jafarzadeh et al., 2017)
*Hypericum perforatum*	**Increase** neutrophil GSH-Px activity, **reduce** lipid peroxidation, apoptosis, and Ca2 + concentrations in MS patients (Naziroglu et al., 2014)

### Artemisia dracunculus

Flavonoids, phenylpropanoids, coumarins, tannins, and essential oils are contained in *Artemisia dracunculus* (*A. dracunculus*) a perennial herb of the Asteraceae (daisy) family, with potent antibacterial, antitumoral, antidiabetic, free radicals scavenging, antioxidant effects. Phenolic compounds and flavonoids, which are the main compounds of *A. dracunculus*, play a key role in the antioxidant activities (Behbahani et al., 2017). The anti-inflammatory effect of the aqueous A. *dracunculus* extract could be due to the inhibition of nuclear factor-kB (NF-kB) signaling (Aglarova et al., 2008) and reduction of secretion of IFNγ and IL-6, as observed in peripheral blood mononuclear cells (PBMC) (Bisht et al., 2021).

Safari et al. (2021) studied the potential use of the aqueous extract of *A. dracunculus* in the treatment of MS and showed that oral administration of the extract to animal models of MS, such as EAE, modulates immune responses, increases the antioxidants serum levels, and significantly reduces IL-17A and IL-23 synthesis in spleen mononuclear cells, and decreases infiltration of the immune cells and demyelination in the EAE mice brain, leading a reduction of severity of neurological deficits and clinical scores and an increase in body weight, suggesting that the compounds, contained in *A. dracunculus*, could be used in the MS treatment.

### Curcuma longa

Curcumin, dimethoxy curcumin, and bisdemethoxycurcumin are polyphenols from *Curcuma longa* and, as antioxidants, protect the brain against various oxidative stressors, acting as a powerful hunter of superoxide anions, showing neuroprotective and anti-aging effects (Bala et al., 2006; Cole et al., 2007).

Many genes that are implicated in the trigger of the immune responses, in the acute phase and inflammatory are regulated at the level of NF-kB transcription. Curcumin inhibits NF-kB activation inducing a down-regulation of specific inflammatory genes, with interesting therapeutic implications in MS (Srivastava et al., 1995; Gachpazan et al., 2021). Several studies reported that the clinical severity of the disease in the EAE model was significantly downregulated by curcumin, which was able to reduce the mRNA expression of IL-17, transforming growth factor (TGF)-β, IFN-γ, IL-6, IL-21, IL-23, signal transducer and activator of transcription (STAT)3, and retinoic-acid-receptor-related orphan nuclear receptor gamma (RORγt) (Kanakasabai et al., 2012), to modulate the CD4 + T cells and toll-like receptors (TLRs) (Chearwae and Bright, 2008; Xie et al., 2009).

Nabiuni et al. (2011) highlight that curcumin is a strong candidate for use in the prevention or treatment of neurodegenerative diseases including MS. Curcumin has bright prospects for MS treatment due to its ability to decrease astrocyte proliferation, improve myelinogenesis, to increase the oligodendrocytes’ activity and differentiation (Xie et al., 2011; Eghbaliferiz et al., 2020; Mohammadi et al., 2021). In a study by Feng et al. (2014) the curcumin effects on apoptosis and mitochondrial injury in the EAE model were evaluated. Results showed that curcumin significantly reduces the number of TUNEL (+) cells in both EAE acute and chronic stages. Also, the expression of caspase (CASP)-3, cleaved caspase-9 (Asp353), and cytochrome complex (Cyt-c) were significantly decreased in EAE mice by curcumin treatment for 7-16 and 30 days, while the Myelin Basic Protein (MBP) expression was significantly increased (Feng et al., 2014).

The potential use of curcumin in the MS treatment was suggested by experiments showing that in lipo-polysaccharide-induced human astrocyte cell (U373-MG), the secretion of metalloproteinase (MMP)-9 involved in the enhancement of the BBB permeability, was reduced by curcumin (Eghbaliferiz et al., 2020), and that, in primary microglia of P3-P6 Sprague Dawley rats or C57BL/6 J mice, curcumin can control the axon degeneration, a final destructive stage in the pathogenesis of MS, reducing NO release, through the JNK phosphorylation pathway (Seyedzadeh et al., 2014; Tegenge et al., 2014).

Moreover, Nataraja and Bright showed that curcumin inhibited EAE decreasing IL-12 production from macrophage/microglial cells, and by inhibition of IL-12-induced tyrosine phosphorylation of Janus kinase 2 (JAK2), tyrosine kinase (TYK2), and STAT 3 and STAT4 transcription factors leads a decrease of T cell proliferation and Th1 differentiation (Natarajan and Bright, 2002). Pretreatment of peripheral blood mononuclear cells from patients with MS with curcumin reduced IL-12 production and enhanced production of IL-10 induced by IFN-β (Fahey et al., 2007). The *in vitro* and *in vivo* studies showed the effectiveness of curcumin against the pathogenetic mechanisms of MS, and although further evidence from randomized controlled trials on curcumin efficacy is required, its role as an effective alternative treatment was suggested (Ghanaatian et al., 2019).

### Ginkgo biloba

The study of Noroozian et al. (2011) showed that 8 weeks of *Ginkgo biloba* (*G. biloba*) treatment can improve memory and QoL in people with MS, and a pilot study on people with MS showed that *G. biloba* determines beneficial effects, reducing inflammation and fatigue, and reversing cognitive impairment (Johnson et al., 2006).

Instead, another exploratory pilot study showed that *G. biloba* causes moderate beneficial effects on selected functional measures (e.g., fatigue) in MS patients (Johnson et al., 2006), and double-blind randomized placebo-controlled trials, were performed to determine if *G. biloba* improves the cognitive performance in MS patients, suggest that may have an effect on interference susceptibility and mental flexibility (Lovera et al., 2007), but that treatment with *G. biloba* (120 mg twice a day for 12 weeks) does not improve their cognitive performance (Lovera et al., 2012).

These contradictory clinical findings could depend on the selection, application, and interpretation methodology of functional outcome measures for testing individuals with cognitive impairment. In a study, Yin et al. (2020) in the *in vitro* cell experiments found that *G. biloba* treatment, inhibits the expression of TLR4, NF-κB, iNOS, IL-1β, and TNF-α, and up-regulating the expression of Arg-1, and neurotrophic factors, significantly regulated the microglia and astrocytes dynamic balance, induced the oligodendrocyte precursor cells (OPCs) generation and promoted myelin production.

Although the precise *G. biloba* action mechanism on microglia and astrocytes has not been extensively investigated, it is supposed that its use may be a novel strategy for MS clinic treatment.

### Cannabis sativa

The *Cannabis sativa* (C. *sativa*) properties have been identified a long time ago. Current studies on the endocannabinoid system allowed the development of new therapeutic targets for the treatment of several diseases such as MS. A double-blind randomized placebo-controlled study pointed out the efficacy of smoking a marijuana cigarette, containing 1.54% A9-tetrahydrocannabinol, in improvement of spasticity, neuropathic pain, urinary complications, and sleep disorders (Greenberg et al., 1994; Pertwee, 2002; Baker and Pryce, 2008).

The neuroprotective cannabinoids effects in MS have been ascribed to the stimulation of CB1R, the most abundant GPCR in the brain, whereas CB2R has been associated with immunomodulatory effects (Al-Ghezi et al., 2019; Mecha et al., 2020).

The Δ-9tetrahydrocannabinol (THC), a *C. sativa* key compound, acts as a partial agonist to the cannabinoid receptors CB1 and CB2 (Mecha et al., 2020), displaying anti-inflammatory and neuroprotective properties, suppressing the IFNγ, IL-17, IL-6, IL-1β, and TNF-α production in the brain and preventing the infiltration of immune cells into the CNS.

Cannabinoid oil extract formulations reduce TNF-α and enhance brain-derived neurotrophic factor (BDNF) production, improving neurological disability scoring in EAE mice (Zhou et al., 2019).

Nichols et al. (2021) demonstrated that early oral administration of cannabidiol reduces EAE severity by suppressing IFN-γ producing CD8^+^ T cells. Cannabidiol treatment is able to increase myeloid-derived suppressor cells (MDSCs) in EAE mice, reduce CXCL9, CXCL10, and IL-1β expression within the CNS, diminish T cell, and inflammatory macrophages infiltration in the CNS, and decrease levels of IL-17 and IFNγ (Elliott et al., 2018; Dopkins et al., 2021). In encephalitogenic T cells of EAE animals, histone methylation signals were differentially enriched in the binding sites of Zinc Finger Protein 143 (ZNF143) and Forkhead Box A1 (FoxA1) transcription factors by cannabidiol, resulting in modulating the expression of the inflammatory genes and clinical symptoms attenuation (Yang et al., 2019). A recent systematic review, by Furgiuele et al. (2021), reported that preclinical evidence in rodent models of EAE strongly supports cannabidiol as an effective immunomodulating and disease-modifying drug.

Many *in vivo* studies performed on MS patients got contrasting data, perhaps due mainly to inadequate therapeutic regimens (Killestein et al., 2003; Centonze et al., 2009; Sorosina et al., 2018; Fragoso et al., 2020; Alijani and Simal-Gandara, 2021). Currently, several cannabis-derived drugs have been tested and approved for medical use and, in some countries, they have been legalized as a therapeutic option for MS-related spasticity and pain.

### Nigella sativa

*Nigella sativa* (*N. sativa*), commonly known as black cumin, has been extensively used in neurodegenerative diseases like Parkinson’s and Alzheimer’s disease because of its antioxidant potential (Fahmy et al., 2014).

***Nigella sativa*** improves the clinical manifestations of EAE reducing the relapse days, improving the daily body weight, increasing locomotor activity, decreasing anxiety, and reducing the neuronal damage in the deep cerebral cortex (Mohamed et al., 2005).

The thymoquinone treatment (1 mg/kg/day), an *N. sativa* key compound, counteract the perivascular cuffing and the mononuclear cells infiltration in the brain and spinal cord, increase the red blood cells glutathione (GSH), and inhibit the NF-kB activation in EAE rat brain and spinal cord, in accord with the clinical sign improvement, decrease the number of reactive astrocytes, the proliferation of the microglial cells and stimulate the complete remyelination detected by brain ultrastructural examination (Mohamed et al., 2005).

A study by Noor et al. showed that in the cerebellum and medulla of EAE rat, the N. *sativa* decreases catalase and increases GSH levels modulating the oxidative balance. Thus, they proposed that *N. sativa* could represent a promising effective agent in both the protection and the treatment of EAE even when the treatment starts after the first clinical signs appearance (Noor et al., 2015).

### Panax ginseng

Flavonoids, triterpenoids, and polysaccharides are among the *Panax ginseng* (*P. ginseng*) active phytochemical compounds for several mental disorders’ treatment, with positive effects due to their antidepressant, antifatigue, and antioxidant properties.

*Panax ginseng* can modulate the immune system in a proinflammation and antiinflammation-balanced manner. The ginsenoside fraction Rb1 can balance Th1 and Th2 cells differentiation and ginsenosides Rd, and Re can improve both the Th1- and Th2-related cytokine release (Rivera et al., 2005; Kang and Min, 2012).

The *P. ginseng* effects on MS are mixed. The study by Etemadifar et al. (2013) showed that the ginseng intake (250 mg/twice daily), significantly improves the quality of life and reduces the level of fatigue in MS patients, while a single center randomized double-blind placebo-controlled crossover pilot study conducted enrolling 56 patients showed that American ginseng extract was safe but ineffective for treating MS fatigue (Kim et al., 2011).

Many studies were conducted on EAE model showing that *P. ginseng*, inducing the immunosuppressive T (Treg) regulatory cells generation and Th1 and Th17 cells suppression, can slow down the EAE progression (Hwang et al., 2011; Lee et al., 2016).

*Panax ginseng* modulates the CD4 + T cells and CD11b + macrophages infiltration into the spinal cord and reduces demyelination and axonal damages other than to reduce the pro-inflammatory factors production such as IFN-γ, IL-17, and TNF-α in both mice models of primary progressive and relapsing-remitting MS (Bing et al., 2016).

Lee et al. (2018) reported that Korean Red Ginseng extract (KRGE) could protect spinal demyelination in the acute EAE model by downregulating the p38 MAPK/NF-κB signaling pathway. The EAE clinical severity can be reduced by treatment with ginsenoside Rd which decreases the permeability of the blood-brain barrier, and endorse the Th2 cell shift (Zhu et al., 2014). The EAE clinical signs decrease by aqueous extract treatment of North American ginseng by inhibition of circulating TNF-α, and CNS immunoreactive inducible nitric oxide (iNOS) (Bowie et al., 2012).

### Zingiber officinale

The Zingiber officinale (Z. *officinale*) root is routinely used as a traditional drug for its antioxidant and anti-inflammatory activities. Ginger and its bioactive compounds could be considered as potential agents to treat MS due to their anti-inflammatory, antioxidant and immunomodulatory properties (Jafarzadeh and Nemati, 2018). Gingerols and their dehydrated derivatives (shogaols) are the major ginger components that exhibit anti-inflammatory effects, the iNOS inhibition, and the cyclooxygenase-2 (COX-2) expression in macrophages, as well as the apoptosis rate reduction in CNS (Ali et al., 2008; Ha et al., 2012). Several studies have reported that ginger extract modulates the IL-17, IL-27, and IL-33 expression in the EAE mice spinal cord, which reduces the IL-23 expression in the spinal cord, and its serum levels. These down-regulate the C-C Motif Chemokine Ligand (CCL)20 and CCL22 expression and their receptors in EAE mice, inhibiting the leukocytes infiltration into the CNS, an essential step in the MS pathogenesis, and improving the clinical symptoms, and the pathological scores of disease (Jafarzadeh et al., 2014, 2015, 2017). It is well known that TGF-β prevents the sensitized T cells’ migration into the CNS, and protects against EAE and MS, while IL-12 may play a major role in EAE development through the induction of both Th1 and Th17 cells differentiation, and both may play a complementary role in the EAE disease pathogenesis (Fletcher et al., 2010). Ginger and its ingredients regulate the Th-1, Th-2, Th-9, Th-17, Th-22, and Treg cell-related immune responses, down-regulating the B cell-related immune responses, modulate the macrophages-related responses, and the inflammatory mediator production and the adhesion molecules expression, interfere with the toll-like receptor (TLR)-related signaling pathways, suppress the inflammasomes and the oxidative stress (Jafarzadeh and Nemati, 2018).

Although additional clinical studies are necessary to determine the most effective doses and their safety, based on these beneficial effects, ginger may be a promising candidate for MS treatment.

### Hypericum perforatum

*Hypericum perforatum* (*H. perforatum*) is used for treating inflammation-related disorders, cancers, and neurodegenerative diseases (Werneke et al., 2004), and for its antidepressant, antioxidant and anti-inflammatory effects can be recommended to MS patients. In fact, in its extract, phloroglucinols, naphthodianthrones, hypericin, pseudohypericin, phenolic compounds, and flavonoids are present, with anti-inflammatory, anti-apoptotic, and antioxidant activities.

The antioxidant content in plant extract is affected by several factors, including extraction procedures (Dresler et al., 2018), while its modulator role, in the treatment of MS, is due to ROS production inhibition (Mojaverrostami et al., 2018).

The study by Naziroglu et al. (2014) showed that the treatment of MS patients with *H. perforatum* decreased until control levels, the lipid peroxidation, apoptosis, and Ca^2+^ concentrations, whereas the plasma glutathione peroxidase (GSH-Px), and serum vitamins A and E values result to be increased. Mesenchymal stem cells derived from adipose tissue (AT-MSCs) are used for the treatment of inflammatory diseases, including MS, and high concentrations of *H. perforatum* extract may improve the AT-MSCs proliferation and their immunomodulatory properties. Thus, *H. perforatum* may be proposed as a supplementary treatment for MS patients who receive MSC cell transplantation (Afsharzadeh et al., 2021).

### Several other herbs and their active compounds

Many other herbs, showing anti-inflammatory and antioxidant properties, have potential therapeutic effects on MS, alleviating the disease severity and reducing the neuropathological changes. Phenolic compounds are the main active chemicals of green tea, comprising epigallo-catechin-3- gallate (EGCG), epicatechin-3-gallate (ECG), epigallocatechin (EGC), and epicatechin (EC), by reduction of IL-1β, INFγ, TNF-α, and iNOS, carry out an antioxidant, and anti-inflammatory activities (Chatterjee et al., 2012).

The EGCG administration to EAE mice decreases brain inflammation and neuronal damage reducing the inflammatory infiltration, proliferation, and differentiation of auto-reactive T-cells in the CNS (Aktas et al., 2004).

Also, the plasma levels of soluble intercellular adhesion molecule-1 (ICAM-1) and CD4 + T-cells expressing C-C chemokine receptor 6 (CCR6) are reduced in EAE mice treated with EGCG (Wang et al., 2012; Sun et al., 2013; Semnani et al., 2017). Recent studies have reported that *Boswellia* spp. possesses neuroprotective properties and is able to boost new nerve networks’ structural formation (Sedighi et al., 2014; Sturner et al., 2014).

The *Ruta graveolens* therapeutic role, an evergreen shrub with a characteristic smell, instead, in the pathological condition of MS, may be linked to its inflammatory and antioxidant properties, in association with the ability to inhibit potassium channels of isolated axons (Bohuslavizki et al., 1992).

In a randomized, double-blind, placebo-controlled clinical study, the modulatory effect of a food supplementation with lemon verbena (*Aloysia citrodora*) has been assessed. The observed data showed a significant reduction of C-reactive protein (CRP) and IFNγ in patients with secondary-progressive MS (Mauriz et al., 2015).

## Medicinal herbs: Adverse effects and potential mechanisms of action

Along with the increased clinical use of herbal medicines, taken in the form of drugs or food, also attention to their safety, quality, appropriate use and the frequency of harmful or unpleasant adverse reactions (ADR), associated with the use of herbal medicine was increased (Shetti et al., 2011; Posadzki et al., 2013). 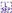
The Taiwan Adverse Drug Reaction Reporting System for Herbal Medicine (TADRRS-HM) database reported the most important causes for adverse reactions to herbal medicine, among which: incorrect processing techniques, improper mixing dosages, harmful interactions between different herbs, wrong methods of decoction, but also the inappropriate indications or dosage, a mismatch between the prescribed formula and the disease pattern can be responsible for adverse effects, other than individual factors such as the individual’s organism characteristics or immune system status, are important. Subjects that consume multiple herbal medicines or formulations that contain multiple components can take risks of herb–herb interactions.

The complexity of the active ingredients of any herbal medicine is further enhanced by the simultaneous use of multiple herbal medicines in one formulation. The properties of herbal medicines can be altered by different processing techniques increasing or altering the therapeutic effect, decreasing toxicity or reducing unwanted adverse effects, and improper processing of herbal medicines can lead to adverse reactions.

Generally, herbal medicines are tolerated satisfactorily, having few mild or moderate adverse effects when used appropriately, but few herbal medicines have undergone randomized clinical trials to determine their safety and quality, and most of the reported clinical studies’ results were not significative due to methodological flaws that made the data of dubious value (duration, use of the blinding, sample size, subject selection, controls adequacy, mode of treatment, use of quantitative outcome measures, and results reports) (Gagnier et al., 2006).

Furthermore, it is not always possible to reproduce the experimental results in a real context. The herbal drug effects seen *in vitro* may be different from those *in vivo* and depend on the species being treated. Finally, in the different pharmaceutical formulations, there may be several active ingredients, in different proportions, extracted and processed with different methods (alcoholic, aqueous extraction, etc.).

A herbal drug may contain many phytochemicals, that could interact synergistically and multi-functionally, making the safety and efficacy assessment very difficult (World Health Organization [WHO], 2004). The quality of medicinal herbs was linked also to chemical composition since sometimes large variations occur within the same species (Randriamampionona et al., 2007) and the presence of secondary metabolites can be linked also to climate, location and parasites other than genetic factors.

In fact, even some herbal drugs, that appear to be helpful, may be harmful intrinsically, by interaction with other medications (Zhang et al., 2015) or extrinsically by factors affecting their quality such as environmental conditions, cultivation and field collection practices, storage, manufacturing or contamination (Fong et al., 2006). The intrinsic toxicity can be conditioned by the variation of the content of the active ingredients present. The chemical constituents of a plant depend on the soil in which they are grown, rainfall and sunshine, the harvest season, the growth stage of the plant during harvest, the diseases afflicting it and the parts that have been harvested. Even in finished products there can be large variations in content from batch to batch and this can cause toxicity.

Many plants produce toxic secondary metabolites as a natural defense that may be harmful to humans. Furthermore, during the harvesting of medicinal herbs, where more than a single species is grown on a particular farm or site, contamination can occur. In an example of contamination, it was found that the adverse reactions were attributed to *Plantago ovata* Forsk. were really caused by *Digitalis lanata* Ehr., a contaminant introduced during the plantain herbs harvest (Slifman et al., 1998).

## Safety of medicinal herbs suggested for the treatment of multiple sclerosis patients

In MS patients were evaluated adverse reactions or toxicities of several medicinal plants such as *G. biloba*, which acts as an antiplatelet agent, and it is important to consider its potential interaction with anticoagulant drugs, as well as its monoamine oxidase (MAO) inhibitory function (Diamond et al., 2000). A clinical trial on 56 MS patients, showed 29 adverse events such as headache, rash, nausea, insomnia, and flu-like syndrome recorded during ginseng administration compared to 26 that occurred during placebo intake (Kim et al., 2011).

*Cannabis sativa* in MS patients is extensively studied, and clinical trials monitoring the safety characteristics of cannabis showed few cases of urinary retention and detrusor sphincter dyssynergia (Vaney et al., 2004). In reverse, the clinical study of *P. ginseng* is very limited, and no serious adverse events were observed in MS patients during follow-up (Etemadifar et al., 2013).

The growing demand for herbal products requires intensive and prolonged agricultural production that needs the use of fertilizers and pesticides. Since “natural” does not necessarily mean “safe,” the effects of pesticide residues’ presence and their quantity must be monitored, because they may represent a serious health risk to consumers. In fact, plant-based drugs may contain pesticide residues, including their metabolites and/or degradation products, which accumulate in the soil and plants during farming (tillage, fumigation, spraying) so becoming a contamination source (Kunle et al., 2012). This makes the safety of herbal products a major public health concern, given the cumulative, additive, or synergistic effects of pesticides on human health.

Unfortunately, at this time many of the plants used in alternative medicine, potentially effective, have not been subjected to the evaluation of toxicity, interference with drugs, and safety. There are no regulated manufacturing standards for many herbal products and some of them have been found contaminated by toxicants or other drugs.

## Medicinal herbs: Quality assurance and quality control

The mechanisms of action and bioavailability of herbal medicines should be determined, and preclinical assessment for safety and efficacy must be performed in animal and *in vitro* models before clinical studies in humans. Cytotoxicity assays (CTAs), that use cell culture, detected how cellular functions may be affected by herbal products. Recently, to predict both helpful and hazardous effects of herbal compounds, DNA sequencing, gene expression, protein, and metabolite profiling were used. If cellular assays are predictive of potential toxicity, the use of animal models allows the evaluation of toxicity signs occurring in response to the gradual increase in the dose of the substance, considering that the biological response could be species-specific and an active ingredient in animals could be completely inactive in humans.

The intricacy of the chemical composition of medicinal herbs and the presence of hazardous agents (pesticides, herbicides, radioelements and microorganisms and heavy metals) is the basis of quality control analyses performed by several methods, such as Nuclear Mass Resonance Spectroscopy (NMR), thin-layer chromatography (TLC), High-Pressure Liquid Chromatography (HPLC), and Liquid Chromatography-Mass Spectroscopy (LC-MS), and the choice of the analytical method, among the many available, is a crucial point that mainly depends on the analytical purposes set. In thin-layer chromatography (TLC), the analytical tool extensively used because of its easiness, relative cheapness and high sensitivity, the compounds may be separated quickly by planar chromatography on silica, cellulose, polyamide or chemically- modified plates, and detected, either directly or after reacting with a specified reagent (Boland et al., 2007). HPLC used to separate, identify, and quantify each component in a mixture has been significantly upgraded, leading to greater separation efficiency, adaptability and speed. Atomic absorption and atomic emission spectroscopies are used for qualitative and quantitative analyses and search for contaminants in herbal drugs (Isaac and Kerber, 2015).

Genetic analyses represent are a new and powerful tool for the reliable identification of a given species, even in a mixture, Allowing to identify traces of DNA in liquid and dry extracts also after repeated processes of dilution/concentration/separation (Novak et al., 2007).

## Pesticide residues in herbal products and regulatory aspects

Often multiple pesticide residues have been found in medicinal herbs. These can also result from cross-contamination from adjacent cultivation sites where pesticides are broadly used or by storage or transport operations. The interactions between several active substances on human health can determine different effects (antagonistic, additive, or even synergistic), such as inducing amplified, irreversible, and even unpredictable damages compared to their single action. International Pharmacopoeias including US^1^ (accessed June 1, 2021), Chinese^2^ (accessed September, 2022), Japanese^3^ (accessed September, 2022), Taiwanese^4^ (accessed September, 2022), European^5^ (accessed June 1, 2021), French^6^ (accessed October 20222021), German^7^ (accessed October 2022), and British^8^ (accessed October 2022), have documented analytical approaches for control and analysis of recognized herbal medicines, and to ensure product safety, current European legislation establishes limits for pesticide residues present in plants used for medicinal purposes through Regulation [EC] 396/2005 (2022) which guarantees “a high level of consumer protection, harmonized Community provisions relating to maximum residue levels (MRL) of pesticides, on or in food and feed products of plant and animal origin.” The MRL limits are calculated on an assessment of the active substance properties and its expected use. The same legal limits, established as tolerance limits, also apply to imported food products to facilitate international trade.

The regulation defines the harmonization of MRLs for products of plant and animal origin, promotes the free circulation of goods, and creates equal conditions of competition between the Member States. The MRL thresholds are set at the lowest possible value consistent with good agricultural practices.

Developing countries, including China, India, and Southeast Asia, are the origin lands and major global suppliers of most of these traditionally used medicinal herbs. High pesticide residues and the levels of their metabolites have been found in foods and supplement especially those from China, the world’s largest producer and exporter of medicinal herbs and food supplements (Nguyen et al., 2010; Wang et al., 2022). Several studies have shown that the production technologies of herbal preparations and supplements can contribute to the elimination of pesticides but, if they are very abundant, they can still be detected after treatment, sometimes reaching concentrations of tens or even hundreds of times higher than the MRL allowed by the European Union (Xiao et al., 2019). In a recent study carried out in China, 1017 medicinal herbs samples, were collected in their main production regions (*Ginseng radix rizoma, Lycii fructus, Houttuyniae herba, Ophiopogonis radix, Alismatis rizoma, Citri reticulatae pericarpium, Fritillaria cirrhosa, Fritillaria unibracteata, Fritillaria przewalskii, Fritillaria delavayi, Fritillaria taipaiensis, Fritillaria unibracteata, Pinelliae rhizoma, Gardeniae fructus*, and *Lonicerae japonicae flos*), have been analyzed to evaluate the residual content of pesticides (Yahia et al., 2016). Pesticide residues were found more frequently in the whole plant or in some parts of it (flowers and fruits). Furthermore, the insecticide detection rate results in higher in the epigeal parts, while plants with roots and rhizomes are more likely to be polluted by organochlorines and growth regulators (Rai et al., 2008). Many medicinal herbs also come from India which, due to its rich biodiversity, is another of the world’s largest suppliers, but it is not always possible to evaluate their quality (Tripathy et al., 2015) Pesticide residues have been also detected in medicinal herbs originating from Brazil (Rodrigues et al., 2007), Iran (Sarkhail et al., 2012), and Egypt (Farag et al., 2011). Several studies have revealed the presence of banned organochlorinated pesticides in American ginseng (Wu et al., 2011) and in commercial ginseng roots (Hayward and Wong, 2009; Wong et al., 2010) used as dietary supplements. Also, dichlorodiphenyltrichloroethane, commonly known as DDT, and its degradation products have been found in some countries (Wang et al., 2022), even though this pesticide has been worldwide banned for agricultural use under the Stockholm Convention (Seventh Expert Group Meeting on DDT – Stockholm Convention (2018)^9^).

In a study carried out by Britt (2000), pyrethroid pesticides have been detected in the roots of *Panax notoginseng* e *Panax ginseng*. These highly liposoluble compounds are easily degraded and excreted by humans (Britt, 2000). In a 2016 study carried out in Saudi Arabia medicinal plants (*Rosmarinus officinals* L., Sa*lvia officinals* L., *Pimpinella anisum* L., *Carum carvi* L., *Cuminum cyminum* L., *Cinnamomum verum* L., *Zingiber officinale Roscoe*, *Camellia sinensis* L.) selected in different markets of Jeddah were analyzed, and pesticide residues were found except in ginger samples. In cinnamon low levels of pesticides were found while in cumin they were very high (Yahia et al., 2016). The different quantity of pesticide residues found between ginger rhizomes and cumin seeds could be due to the different parts of the plant used. The ginger rhizomes have moisture content higher than 80% compared to 6% of cumin seeds, in which there is a high content of volatile oil instead. The oil content in cumin seeds could play a fundamental role in the solubility of pesticides, compared to ginger rhizomes containing a lot of water, because many pesticides are liposoluble (El-Ghorab et al., 2014). In another study carried out in 2020 in Poland, 104 samples of plant matters (thyme herb, savory, sage, rock rose, marjoram, horsetail, oregano, basil, linseed, licorice roots, valerian, lovage, echinacea, and chamomile flowers, and fruits of fennel and cumin) have been analyzed (Grażyna, 2020). The results showed that 72.1% of plant matter samples from eastern Poland crops contained pesticide residues. In 11.5% of the cases has been found that the maximum permissible concentrations exceeded, particularly in the thyme samples (approx. 66%) compared to the other groups of analyzed plants. The causes of toxicity in medicinal plants are attributable to the concentration of metals in soil, air, and water and various contaminants such as pesticides. The growing demand for medicinal herbs requires greater agricultural production and the use of chemicals such as fertilizers and pesticides are especially relevant in intensive farming. Furthermore, the presence of residues of pesticides in medicinal herbs may also be due to cross-contamination from cultivation sites where pesticides are applied or during storage or transport. Combinations of several classes of pesticides featuring additive or synergistic interactions are often used to prevent crops from the destructive effects of pests and to enhance crop productivity. Multi pesticide residues (including fungicides and herbicides) have been detected in medicinal herbs (Tong et al., 2014).

## Pathogenic mechanism of pesticides in neurodegeneration

Over 45% of neurotoxic chemicals are pesticides, commonly found in the environment and in food. The pesticide’s effects on human health can be acute, with symptoms like fatigue, headache and muscle aches, skin discomfort, rash, poor concentration, weakness, circulatory problems, and dizziness (British Crop Production Council [BCPC], 2022). Symptoms may not appear for a long time and have late manifestations. Studies have revealed the correlation between pesticide use and sarcomas, multiple myelomas, prostate cancer, pancreas, lung, ovary, breast, testicular, liver, kidney, bowel, and brain tumors (Alavanja, 2004). Based on epidemiological studies, exposure to pesticides was considered an important risk factor for neurological disorders, including Parkinson’s disease, Alzheimer’s disease, and MS (Yadav et al., 2016), and the most frequent effects related to organophosphate exposure have been listed in the Table 2.

**TABLE 2:** Different types of pesticides to the neurodegenerations risk for human (modified from Kori et al., 2018).

**Organophosphate (chronic exposure)**		
Occupational exposure (farm workers)	Genetic tendency to a less efficient detoxification of paraoxonase, an enzyme detoxifying the organophosphate pesticides Neurological impairment (attention, remembrance, reaction time and deficits in neurobehavioral performance) Delayed polyneuropathy Worse performance in neuropsychological functions (attention, reasoning, memory, perception, visuomotor skills, expressive language and motor performance) Neurological symptom (dizziness, sleepiness and headache) Headache, giddiness, ocular symptoms and Paraesthesia. Reduced motor conduction velocity and serum AChE levels	Bonvicini et al., 2010 Rohlman et al., 2007 Lotti and Moretto, 2005 Salvi et al., 2003; Roldan-Tapia et al., 2005 London et al., 1998 Misra et al., 1988
Occupational and residential exposure	Some neurological and neuropsychiatric effects on adults (ADHD, anxiety, depression disorder) Reduce in the verbal memory, motor speed and motor coordination Significantly higher anxiety and depression in exposed population Lower neurobehavioral performance like learning test, symbol-digit, reaction time Slower response on neuropsychological performance like processing speed, attention Serum acetylcholinesterase was significantly lower, neurological abnormalities Headache, hypertension, anxiety and depression disorders High depressive symptoms, headache and dizziness Sensory and vibration thresholds were higher, neurological symptoms	London et al., 2012 Starks et al., 2012 MackenzieRoss et al., 2010 Rothlein et al., 2006 Stephens and Sreenivasan, 2004 Farahat et al., 2003 Salvi et al., 2003 Stallones and Beseler, 2002 Pilkington et al., 2001
Occupational as well as accidental exposure	Acute and chronic OP exposure is associated with affective disorders Suicide rates are high in farming populations	London et al., 2005
**Organochlorine and pyrethroid (chronic exposure)**		
Occupational exposure (farm workers)	Deterioration in neurocognitive performance Parkinson’s disease Alteration in hematological parameters Abnormal cranial nerve function, and motor strength	Hansen et al., 2017 Elbaz et al., 2009 Lu, 2009
Occupational and residential exposure	Neurotoxic properties Autism spectrum disorders (ASD), neurodevelopmental disorders Acute poisoning and alterations of the digestive, neurological, respiratory, circulatory, dermatological, renal, and reproductive system Alzheimer’s disease Risk of Alzheimer’s disease Decrease in Mental developmental Index scores	Su et al., 2016 Cheslack-Postava et al., 2013 Roberts et al., 2007 Payan-Renteria et al., 2012 Singh et al., 2012; Kim et al., 2015 Richardson et al., 2011 Eskenaz et al., 2006
**Chronic exposure of mixture of pesticides (organophosphate, carbamate, organochlorine, and pyrethroid)**		
Occupational exposure (farm workers)	Higher anger-hostility, depression-dejection, tension-anxiety and lower vigor-activity	Zhang et al., 2016
Occupational and residential exposure	Neurotoxic symptoms Depressive symptoms Somatization, obsessive–compulsiveness, interpersonal sensitivity, depression and anxiety Association between pesticide exposure and depression More inhibition of cholinesterase activity Respiratory, eye disorders. Inhibition of cholinesterase activity and central nervous system problems	Motsoeneng and Dalvie, 2015 Beard et al., 2013 Wesseling et al., 2010 Beseler and Stallones, 2006 Smit et al., 2003 Ohayo-Mitoko et al., 2000

The pathogenic mechanism of pesticides is different and often not well specifically classified. The EPA’s ToxCast program identified 976 chemicals that were tested on mouse astrocytes. Because they trigger inflammatory signals in these cells, 75 of them have been selected. Exposure to pesticides causes alteration of the inflammatory pathway (Nrf2/NF-kB, MAPKs/PI3K, and Akt/GSK3β) reducing NF-E2–related factor (Nrf)2, increasing NF-kB, and opening voltage-gated Ca2 + channel of the neuron’s plasmatic membrane (Silver et al., 2017), leading to oxidative stress, neuronal apoptosis e reduction of serotonin.

The organophosphorus pesticides (OPs), the highest sources of contamination for humans are alimentary ingestion and professional exposures, are well known for their neurotoxicity due to the irreversible inhibition of acetylcholinesterase. OPs down-regulate the expression and the activity of several cholinergic components such as nicotinic and muscarinic cholinergic receptors (nAChR, mAChR), Choline acetyltransferase (ChAT), Acetylcholinesterase, butyrylcholinesterase and vesicular ACh transporter (AChE, BChE, and VAChT), thus leading to increased pro-inflammatory and decrease anti-inflammatory cytokines production. Since cholinergic components are express in many cells type and each of these cells is a possible target of OPs, both acute and chronic exposure to OPs may be related to the development of chronic neurodegenerative disorders, as well as allergies, or immunosuppression phenomena. Thus, the cholinergic components could be helpful biomarkers in the monitoring of populations exposed to pesticides.

In synapses, inhibition of acetylcholinesterase provokes acetylthiocholine accumulation resulting in muscarinic and nicotinic receptor over activation, and nerve function loss (Galloway and Handy, 2003). Inhibition of AChE moves to high levels of ROS, which is due to the inhibition of oxidative phosphorylation. Due to disruption in oxidative phosphorylation, mitochondrial dysfunction was reported following exposure to pesticides. Decreased expression of BCl2 and increased expression of Bax, Caspases-3, 9, JNK1, 2, TrkA/p75 have been detected after pesticide exposure. Also, the cyclic AMP-protein kinase signaling pathway that influences the expression and function of nuclear transcription factors such as c-fos, p53, AP-1, Sp1, and CREB (Ca2 + /cAMP response element-binding protein) involved in the transition from proliferation to differentiation of neural cells is impaired by organophosphates (Costa et al., 2008).

Studies on organophosphorus pesticides have focused the attention on neurons, which are considered to be the primary target for neurotoxicity (Won et al., 2001; Levin et al., 2002), but recently also glial cells were suggested as the target of organophosphorus pesticides. In fact, the increased glial fibrillary acidic protein (GFAP) immunoreactivity, inhibition of replication/differentiation and induction of oxidative stress in C6 glioma cells, and of glial development *in vivo*, were reported (Garcia et al., 2001, 2002; Qiao et al., 2001; Zurich et al., 2004). Glial cells that provide nutrition to the neurons and provide a link with the immune system, responding to damage by acting as scavengers of pathogens and neuronal debris, were more vulnerable than neurons to organophosphorus pesticides. Neurons-glial cell signaling and metabolic interactions are required for the development and maintenance of brain structures and functions. Brain injury causes astrogliosis, an early marker of neurotoxicity, characterized by an increased expression of GFAP and the release of bioactive molecules that can modulate the neurotoxic effects of organophosphorus pesticides (Aschner et al., 2002). Prenatal exposure to organophosphates is associated with neurodevelopmental disturbances. The study by Young et al. (2005) points out the association between increasing total concentrations of organophosphate metabolites in the mother’s urine and abnormal reflexes in neonates.

*In vitro* studies showed increased inflammatory responses in neural cells repeatedly exposed to low levels of organophosphates. Inflammation occurs in the brains of patients with PD, as part of the cause of the disease and not as the effect of the disease and was linked with impaired learning and memory. Thus, the evaluation of the link between pesticides and neurodegeneration was focused on PD, pointing out that occupational exposure to organophosphorus pesticides increases PD risk by induction of intraneuronal oxidative stress and damage, ROS-dependent oxidation of lipids and proteins mitochondrial dysfunction, ATP deficits, impaired unfolded protein response, neuroinflammation, and metabolic disruption (Fitsanakis et al., 2002).

Carbamate pesticides, such as aldicarb, carbofuran, and ziram, are another class of chemical pesticides used on crops, that cause reversible carbamylation of AChE allowing accumulation of acetylcholine, and were related with neuronal damages, oxidative stress, increases the risk for dementia and Parkinson-like neuropathy (Türk Börü et al., 2018).

Both epidemiologic and experimental studies have evidenced that pesticides exposure affects central nervous systems, probably via epigenetic modifications (Yu et al., 2021), such as up-regulation of miRNA-181 that drives the neuronal cells apoptosis as observed by Zhao et al. (2019) in a neuroblastoma cell line. miRNA-181 up-regulation leads to increased oxidative stress, altered neurotransmitter status, neuroinflammation, neurodegeneration, and neuronal apoptosis. Experimental studies have reported that exposure to dieldrin, an organochlorine pesticide, during the development of C57BL/6 mice alters DNA methylation, which contributes to late-life PD development (Pouchieu et al., 2018; Kochmanski et al., 2019).

Although there have been very few studies, most have found a significant association between AD and occupational pesticide exposure, and, compared to PD, there is more consensus that pesticide exposure increases the risk of AD (Laske et al., 2004). The case-control study conducted by Mar et al. (2018) in the USA, suggest that exposure to rodenticides, weed control products, plant/tree insect, or disease control products, during early childhood is associated with the risk of developing pediatric-onset MS.

## Pesticides and multiple sclerosis

Currently about 25 million workers each year suffer unplanned pesticide poisoning, due to inhalation or skin absorption. According to epidemiological studies, human exposure to pesticides could also constitute a significant risk factor for neurological disorders such as Parkinson’s disease, Alzheimer’s disease, and MS (Parrón et al., 2011; Baltazar et al., 2014).

An increased risk of MS was observed in the agricultural sector workers, exposed to pesticides suggesting their possible role in the pathogenesis of MS for the well-known neurotoxic effect (Horwitz et al., 2013; Oddone et al., 2013; Magyari et al., 2014), despite the difficulties encountered both in the data acquisition (duration, frequency, and intensity of exposure of the subjects, lifestyle habits) and in the interpretation of the results (Marrie, 2004). Identifying pesticides as risk factors is challenging, given the high number of different pesticides and other factors that we are every day exposed to. The study by Graves et al. (2017) showed that pesticide exposure in the household, in the perinatal period, can be associated with an increased risk of pediatric-MS onset. The study of Costa et al. (2008) shows that during the mixing, loading, and distribution of pesticides and in any subsequent crop handling and harvesting operations, especially in the absence of adequate protective devices, the worker is subject to the risk of acute and chronic exposure. In a study conducted in Andalusian health districts, on 17,429 total medical records selected in areas with high and low environmental exposure to pesticides, collected between 1998 and 2005, it was observed that the risk of developing neurodegenerative diseases, including MS, was significantly higher in districts with greater pesticides use (Parrón et al., 2011). In another study carried out in three Turkey cities, one in the Mediterranean region (Gazipaşa) and two in the Black Sea region (Artvin and Ordu), 26 patients with MS were detected. Most of them are in Gazipaşa (Turkey), where the main source of income is greenhouse vegetables and fruit cultivation, with high use of pesticides, although the city of Ordu is more industrialized. In Artvin, a city set in an uncontaminated natural environment, the incidence is much lower (Türk Börü et al., 2018). Wheeler et al. (2019) published the results of a study on environmental exposure to chemicals and pesticides, stating that it may be a risk factor in the development of neurodegenerative diseases. Mowry et al. (2018) in California, carried out a study on 2,310 people with MS and 4,819 controls, by questionnaires concerning lifestyle (diet, caffeine consumption, and cigarette smoking), and environmental and occupational exposures to pesticides or herbicides. No significant associations related to MS have been observed, but researchers, using a machine-learning algorithm, identified a higher probability of contracting MS in male subjects carrying the MS-associated human leukocyte antigen (HLA)DRB1*1501, exposed to pesticides (Mowry et al., 2018).

In the axonal loss, which is considered to be the main cause of permanent clinical disability in MS, key elements are inflammation, mitochondrial alteration, upregulation of sodium channels and glutamate excitotoxicity. The inflammatory process present in MS causes high levels of reactive oxygen and nitrogen species, which may lead to alteration of mitochondrial function (energy deficit, apoptosis), with alteration in glutamate (Glu) homeostasis with excessive calcium influx, which may play an important role in the pathogenesis of MS causing axonal and neuronal damage and promoting disease progression (Friese et al., 2014). Thus, inflammation, free radical production, lipid peroxidation, and mitochondrial dysfunction are target by which most pesticides, including organophosphates and herbicides, induced neuronal degeneration and tissue damage in MS.

Exposure to relatively low doses of pesticide may affect brain cells and cause loss of neurons in specific regions of the brain by induction of free radical production or lipid peroxidation, with subsequent cognitive, memory, attention decline, as well as motor function impairment in MS (Franco et al., 2009). OPs in T cells, microglia/macrophages, astrocytes, oligodendrocytes, and neuron in the CNS of MS patients reduces Nrf2 and increases NF-kB, and may promote the release of inflammatory cytokines. One of the most important effect of OPs is the inhibition of AChE, causing accumulation of acetylcholine at cholinergic synapses with excessive activation of muscarinic and nicotinic receptors and over-stimulation of cholinergic neurons, and also the production of cytokines and other pro-inflammatory molecules (Camacho-Pérez et al., 2022).

Organophosphorus pesticides increasing the mitochondrial calcium uptake, impairs complex I and II function and decrease electron transfer activities of cytochrome oxidase, and the activities of mitochondrial electron transfer enzyme, are responsible of lipid peroxidation, of 8-hydroxydeoxyguanosine formation and protein and mtDNA oxidation (Leung and Meyer, 2019).

Moreover, chronic OP exposure has the potential of generating reactive oxygen species and/or impairing cellular, leading to oxidative stress. In summary, chronic low-level exposure to OPs impairs mitochondrial bioenergetics resulting in apoptotic neuronal degeneration (Kaur et al., 2007; Wani et al., 2011). Ops also induce activation of glutamatergic neurons with glutamate release that leads to activation of *N*-methyl-D-aspartate (NMDA) receptors (Milatovic et al., 2005).

The study of Wheeler et al. (2019) showed that Linuron, a herbicide banned in Europe since 2017 due to its potential health risks, can promote immune-mediated proinflammatory signals in the mice’s central nervous system causing MS-like symptoms. In CNS samples from MS patients increased X-box binding protein 1 (*XBP1)* and increased astrocyte-specific inositol-requiring enzyme-1α (IRE1α) activation were detected, confirming their contribution to MS pathology (Wheeler et al., 2019), and Linuron activating signaling via sigma receptor 1, IRE1α, and XBP1 amplify proinflammatory response and drive pathogenic activities in astrocytes. Indeed, in astrocytes, Linuron synergized with IL-1β and TNFα to induce unfolded protein accumulation and boosted pro-inflammatory gene expression such as Nos2, IL6, CSF2, and CLl2 (Hedström et al., 2018).

Brain tissue samples from MS patients have been analyzed, and elevated levels of inflammatory responses, like in mouse astrocytes exposed to Linuron, were found (Wheeler et al., 2019). Furthermore, the Hexachlorobenzene pesticide, which was widely used until 1965, through the activation of the integrin-linked kinase (ILK) signaling, modulates the expression of E-cadherin, which plays an important role in BBB dysfunction, facilitating the leukocytes massive influx into the brain parenchyma, which in turn induces irreversible demyelination, tissue damage, and axonal dysfunction (Agency for Toxic Substances and Disease Registry [ATSDR], 2022).

The organic solvents’ precise effect on DNA methylation warrants further investigation but is known that in MS patients the immune regulatory gene protein tyrosine phosphatase (SHP)1 promoter region is highly methylated, facilitating the immune cell infiltration into the brain (Kumagai et al., 2012).

## Conclusion and future directions

The employment of herbal medicines, often cheaper and less expensive than pharmaceuticals, continues to expand rapidly across the world. Epidemiological studies indicated that herbal medicines, by reduction of inflammation, oxidative stress, and neuronal damage, could be a promising strategy to treat MS.

Unfortunately, the increased use of medicinal herbs requires an intensification of agricultural production, and this has led to the extensive use of pesticides, especially in countries where the legislation to regulate their use is lacking.

In this overview, we have analyzed epidemiological studies to highlight the risk of MS in farmers exposed to pesticides during the pesticide spray solutions preparation and application. These studies presented several limitations, in fact, they did not adjust for potential confounding variables such as genetic factors, sun exposure, vitamin D intake. Furthermore, the translation of an *in vitro* biological and pharmacological effect on *in vivo* effects cannot always be taken for established, due to individual differences or other variables such as the type of plant extract and methods of processing. Thus, coming investigations will take these limitations into account and the study of gene-environment interactions may aid in identifying the potential role of pesticides in the risk for MS onset. Future studies will have to investigate the mechanisms by which pesticides get rid of insects and which can be involved in the increase in the risk of MS, to highlight the new potential biological target of MS.

In conclusion, to discover novel therapeutics composed of multiple herbal compounds and to protect human health, herbal medicines should be cultivated and/or collected in an uncontaminated environment, reducing the use of pesticides and avoiding the presence of chemicals residues and of toxic elements to ensure farmers’ and consumers’ safety. The exhaustive quality control of herbal medicines conducted by chemical, pharmacological, medical and even statistical interdisciplinary investigations is necessary to ensure the safest use, treatment efficacy and the required potency of the products that will benefit people and society by providing means of well-being. Furthermore, since in many developing countries unregistered and poorly regulated herbal products may be sold with little or no limitation, international regulatory policies are needed which take into account also of the new knowledge on the impact of pesticides on neurodevelopment and neurodegeneration.

## Author contributions

MR, LC, and EM provided the idea for the review. EC, MR, and EM searched the literature, prepared the figures, and wrote the manuscript. LC and MDL participated in reviewing the literature. All authors contributed to the article and approved the submitted version.

## Footnotes

1
https://www.usp.org

2
https://www.usp.org/products/chinese-pharmacopoeia

3
https://www.pmda.go.jp/english/rs-sb-std/standards-development/jp/0009.html

4
https://www.mohw.gov.tw/lp-3690-2.html

5
https://www.edqm.eu/en/european-pharmacopoeia-ph-eur-10th-edition

6
https://ansm.sante.fr/documents/reference/pharmacopee/la-pharmacopee-francaise

7
https://www.bfarm.de/DE/Arzneimittel/Arzneimittelzulassung/ZulassungsrelevanteThemen/Ar_neibuch/_node.html;jsessionid=3EF7531E89D7D725DF8E 0192A3627F09.1_cid319

8
https://www.pharmacopoeia.com/

9
http://chm.pops.int
